# Rapid Changes in the Phytoplankton Community of a Subtropical, Shallow, Hypereutrophic Lake During the Rainy Season

**DOI:** 10.3389/fmicb.2021.617151

**Published:** 2021-03-09

**Authors:** Osiris Díaz-Torres, José de Anda, Ofelia Yadira Lugo-Melchor, Adriana Pacheco, Danielle A. Orozco-Nunnelly, Harvey Shear, Carolina Senés-Guerrero, Misael Sebastián Gradilla-Hernández

**Affiliations:** ^1^Centro de Investigación y Asistencia en Tecnología y Diseño del Estado de Jalisco, A.C., Unidad de Servicios Analiticos y Metrologicos, Guadalajara, Mexico; ^2^Departamento de Tecnologia Ambiental, Centro de Investigación y Asistencia en Tecnología y Diseño del Estado de Jalisco, A.C., Zapopan, Mexico; ^3^Tecnologico de Monterrey, Escuela de Ingenieria y Ciencias, Monterrey, Mexico; ^4^Department of Biology, Valparaiso University, Valparaiso, IN, United States; ^5^Department of Geography, Geomatics and Environment, University of Toronto-Mississauga, Mississauga, ON, Canada; ^6^Tecnologico de Monterrey, Escuela de Ingenieria y Ciencias, Zapopan, Mexico

**Keywords:** cyanobacteria, microalgae, physicochemical and environmental parameters, limiting nutrient, microcystin, Lake Cajititlán, fish mortality

## Abstract

Lake Cajititlán is a small, shallow, subtropical lake located in an endorheic basin in western Mexico. It is characterized by a strong seasonality of climate with pronounced wet and dry seasons and has been classified as a hypereutrophic lake. This eutrophication was driven by improperly treated sewage discharges from four municipal wastewater treatment plants (WWTPs) and by excessive agricultural activities, including the overuse of fertilizers that reach the lake through surface runoff during the rainy season. This nutrient rich runoff has caused algal blooms, which have led to anoxic or hypoxic conditions, resulting in large-scale fish deaths that have occurred during or immediately after the rainy season. This study investigated the changes in the phytoplankton community in Lake Cajititlán during the rainy season and the association between these changes and the physicochemical water quality and environmental parameters measured in the lake’s basin. *Planktothrix* and *Cylindrospermopsis* were the dominant genera of the cyanobacterial community, while the Chlorophyceae, Chrysophyceae, and Trebouxiophyceae classes dominated the microalgae community. However, the results showed a significant temporal shift in the phytoplankton communities in Lake Cajititlán induced by the rainy season. The findings of this study suggest that significant climatic variations cause high seasonal surface runoff and rapid changes in the water quality (Chlorophyll-*a*, DO, NH_4_^+^, and NO_3_^–^) and in variations in the composition of the phytoplankton community. Finally, an alternation between phosphorus and nitrogen limitation was observed in Lake Cajititlán during the rainy season, clearly correlating to the presence of *Planktothrix* when the lake was limited by phosphorus and to the presence of *Cylindrospermopsis* when the lake was limited by nitrogen. The evidence presented in this study supports the idea that the death of fish in Lake Cajititlán could be mainly caused by anoxia, caused by rapid changes in water quality during the rainy season. Based on our review of the literature, this is the first study on the phytoplankton community in a subtropical lake during the rainy season using high throughput 16S rRNA and 18S rRNA amplicon sequencing.

## Introduction

Lake Cajititlán is a small, shallow subtropical lake located in an endorheic basin in the municipality of Tlajomulco de Zúniga in the state of Jalisco, Mexico at 1,552 m a. s. l. (Limón-Macias et al., 1983). It represents an important regional water resource for the harvesting of endemic fish species such as: charal (*Menidia Grandocule*), tiro (*Goodea atripinnis*), popocha (*Algansea popoche*), and pintitas (*Posiliopis infans*) (Rosales-Figueroa, 1994; Caro-Becerra et al., 2007; Luján-Godínez et al., 2014; Vizcaíno-Rodríguez et al., 2018). The basin of Lake Cajititlán is characterized by strong seasonality of climate with pronounced wet and dry seasons. Agriculture is the main economic activity within the basin. However, most of the agriculture is rainfed, which means that fertilizers are used during the rainy season, often in excessive amounts. These agricultural practices are one of the principal sources of nutrient contamination leading to cultural eutrophication in the Lake (de Anda et al., 2019a). The poor water quality in the Lake is also due to partially treated sewage that is discharged into the lake from three municipal WWTPs; these discharges frequently do not meet the water quality standards required by federal regulations (de Anda et al., 2019b). As a result of this nutrient pollution, the Lake has been classified as hypereutrophic (de Anda et al., 2019a). This process of cultural eutrophication is exacerbated by the endorheic nature of the Lake (IIEG, 2018; de Anda et al., 2019b).

Previous studies have demonstrated the water quality of Lake Cajititlán, as measured by an ecosystem-specific water quality index developed for this lake, consistently reached its lower values during and immediately after the wet season (June-September) for a monitoring period of 9 years (2009–2018) (Gradilla-Hernández et al., 2020b). Several episodes of sudden, large-scale fish mortality have been reported since 2013, mainly during or immediately after the rainy season (Gradilla-Hernández et al., 2018). During this period, runoff from agricultural land and discharges of partially treated wastewater mixed with rainwater result in a large input of nutrients, organic matter, and other pollutants to the lake, causing phytoplankton blooms. As a result, high rates of dissolved oxygen (DO) consumption during the night, have led to episodes of anoxic (zero dissolved oxygen) or hypoxic (low dissolved oxygen) conditions. These conditions are largely responsible for the large-scale fish mortality (Gradilla-Hernández et al., 2018, 2020a,b; de Anda et al., 2019b).

Phytoplankton are the autotrophic component of the planktonic community and therefore the base of the trophic network in aquatic ecosystems. Phytoplankton include photosynthetic prokaryotic (cyanobacteria) and eukaryotic (microalgae) organisms that live near the surface of the water column, where they can capture the necessary light to support photosynthesis (Rouco, 2011). Phytoplankton abundance and distribution in aquatic systems depend on environmental and physicochemical factors, such as nutrient availability (phosphorus and nitrogen) (Mur et al., 1999; Rouco, 2011), light intensity (Rouco, 2011; Su et al., 2014), temperature (Bormans et al., 2004; Rouco, 2011; Zhang et al., 2016), water clarity (turbidity) (Zhang et al., 2016) and the abundance of other planktonic organisms or predators (Rouco, 2011), as well as their characteristic ecophysiology (e.g., growth rate) (Mur et al., 1999). Phytoplankton blooms impact aquatic ecosystems by depleting oxygen at night and reducing light penetration (Ssebiyonga et al., 2013). In addition, several cyanobacterial genera (*Microcystis*, *Anabaena*, *Planktothrix*, *Oscillatoria*, *Anabaenopsis*, *Nostoc*) produce a group of peptide toxins, known as microcystins (World Health Organization (WHO), 1999; Kaebernick and Neilan, 2001). These cyanotoxins may be absorbed in fish through their gills, or through diet, accumulating in organs, resulting in major damage to the liver and kidney (Lance, 2008), as well as causing cell damage and death through the inhibition of phosphatases (Yoshizawa et al., 1990; Fu et al., 2005).

Tropical and subtropical regions display specific sensitivities to eutrophication because of their climatological attributes. High rainfall in these regions may enhance nutrient runoff from agricultural areas to surficial waters (Cunha et al., 2013). In these regions, nutrient contamination is more strongly oriented toward nitrogen, the most likely limiting nutrient in tropical and subtropical lakes. Primary production in tropical and subtropical lakes is sustained throughout the year as a result of higher temperatures, as opposed to temperate lakes, where the productive seasons are spring and summer (Talling, 1992; Cunha et al., 2013). The limnology of temperate regions has been increasingly focused on the changes in the phytoplankton communities during different seasons (Lenard and Wojciechowska, 2013; Wojciechowska and Lenard, 2014; Hampton et al., 2015; Özkundakci et al., 2015; Kalinowska and Grabowska, 2016; Grosbois et al., 2017; Lenard et al., 2019; Wei et al., 2020). Yet, there are few studies on the temporal dynamics of phytoplankton during different seasons in tropical or subtropical shallow lakes, and even fewer studies that examine the rainy season (Lewis, 1990; Idumah and Ugwumba, 2013).

In comparison to culture-based studies, high throughput sequencing (HTS) can detect a large majority of microbial taxa present. This helps to generate a deeper understanding when comparing populations of phytoplankton (Falconer and Humpage, 2005; Brooks et al., 2015; Tragin et al., 2017). In this study, we have used HTS to assess the phytoplankton community during the rainy season in a eutrophic subtropical and shallow lake. This was accomplished by targeting two hypervariable regions: V3–V4 of the 16S rRNA gene and V4 of the 18S rRNA gene. Based on our review of the literature, this is the first study on the phytoplankton community in a subtropical lake during the rainy season using 16S rRNA and 18S rRNA amplicon HTS.

The objective of this study was to analyze the phytoplankton dynamics during the rainy season in Lake Cajititlán, which has a pronounced hot-dry season (February–May) and a wet season (June–September), as well as strong anthropogenic inputs of pollutants. Additionally, we sought to determine how physicochemical and environmental factors associate with these variations. A deeper understanding of these elements will contribute to our understanding of the impact of seasonality on the water quality of these types of lakes.

## Methodology

### Study Site

**FIGURE 1:**
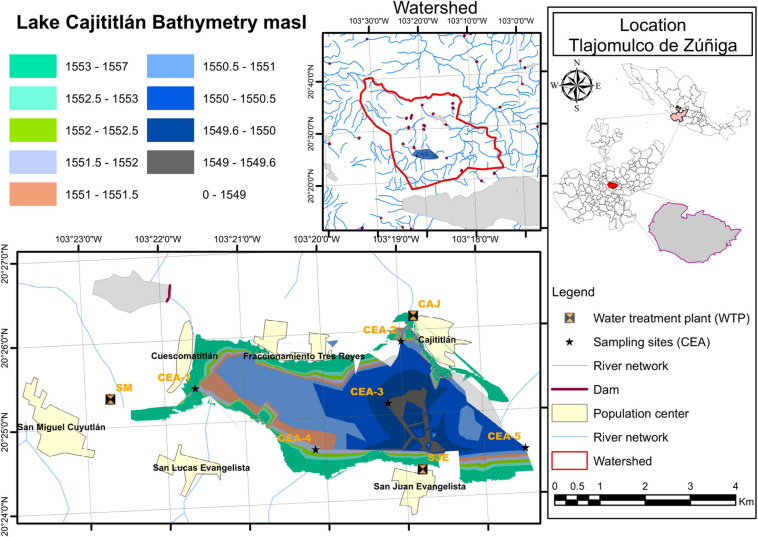
Location of the sampling stations in Lake Cajititlán.

Lake Cajititlán has an annual average surface area of 17.44 km^2^, a maximum depth of 3.87 m, and an average storage volume of approximately 70.89 hm^3^ (de Anda et al., 2019a). According to Lewis (1983), it is classified as a warm polymictic lake. An important feature of the Lake is that it is in an endorheic basin surrounded by small hills. The area of the basin is approximately 201.8 km^2^ (de Anda et al., 2019a; Figure 1). Three seasons generally occur in this basin (i): the hot-dry season (February–May), the wet season (June–September), and the cold-dry season (October–January) (Gradilla-Hernández et al., 2020a).

### Characterization of the Annual Behavior of Climate Data and the Water Quality Features of Lake Cajititlán

Precipitation rates (mm), evaporation rates (mm) and maximum/minimum air temperatures (°C) from both 2018 (when the sampling was performed) as well as historically (from 1998 to 2019) were retrieved from of the National Water Commission of Mexico (“CONAGUA” COMISIÓN NACIONAL DEL AGUA). These measurements were made at a climatological station (ID # 00014072) located 20 km from Lake Cajititlán. These data were analyzed to characterize the climate of this subtropical region. Furthermore, total nitrogen (TN) and total phosphorus (TP) (mg/L) values were retrieved from the State Water Commission (Spanish acronym CEA) data repository for the same five sampling points as used in this study (CEA-1, CEA-2, CEA-3, CEA-4, and CEA-5) at a depth of 0.8 m (Figure 1). These data were obtained as a time series with monthly periodicity from September 2009 to April 2019 (CEA-Jalisco 2019).

**FIGURE 2:**
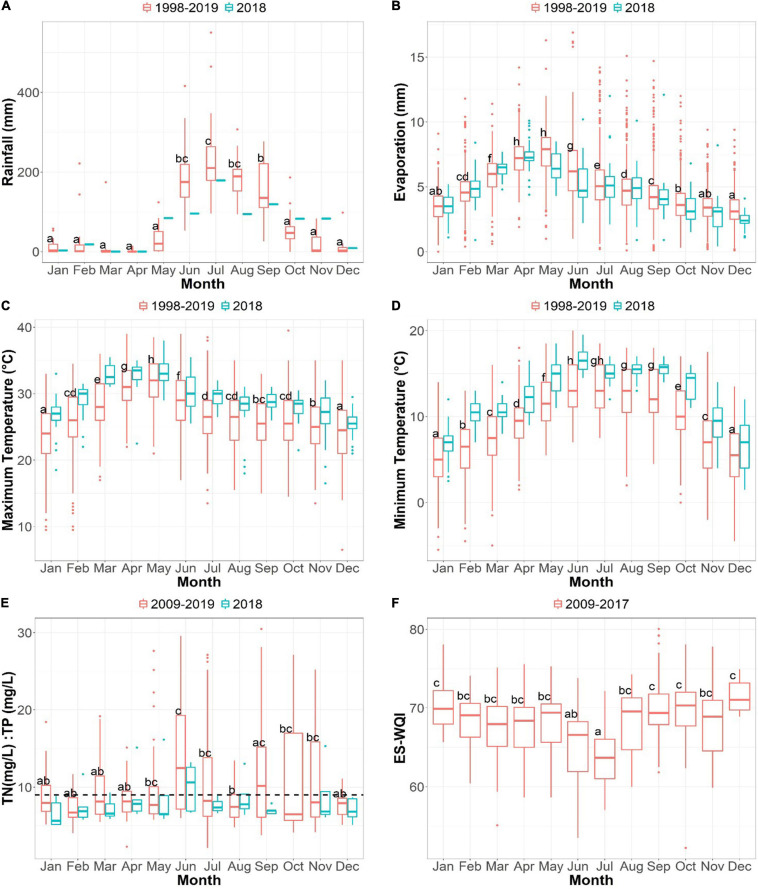
Annual behavior of the mean temperature (°C) (maximum and minimum), rainfall (mm), and evaporation (mm) of Lake Cajititlán over a 21 years period (1998–2019). Annual behavior of the ecosystem-specific water quality index (ES-WQI) of Lake Cajititlán in the period 2009–2017. Annual behavior of the mean TN:TP ratio in Lake Cajititlán over a 10 years period (2009–2019). **(A)** Rainfall (mm). **(B)** Evaporation (mm). **(C)** Maximum temperature (°C). **(D)** Minimum temperature (°C). **(E)** TN:TP ratio (mg/L). **(F)** ES-WQI.

During our field sampling, physicochemical parameters were measured once per month during the rainy season (July–September) to assess the water quality of Lake Cajititlán. Five sampling points (CEA-1, CEA-2, CEA-3, CEA-4, and CEA-5) (Figure 1) and two monitoring depths (80 cm and interstitial) were selected. These sampling points corresponded to the sites established by the CEA to monitor the water quality of the lake (Figure 1). This study period (July–September) was chosen because, historically, the greatest variations in rainfall and TN:TP ratio occur in this season (Figures 2A,E), as well as the lowest average values of the ecosystem-specific water quality index of Lake Cajititlán (Figure 2F; Gradilla-Hernández et al., 2020b). Only one measurement (a total of 30 measurements) was taken for each physicochemical parameter, using two previously calibrated environmental probes (6600 and 6829 V2 YSI^®^ a xylem brand) (YSI, 2009). The following physicochemical parameters were analyzed: dissolved oxygen (DO), water temperature (WT), electrical conductivity (EC), turbidity, pH, oxidation-reduction potential (ORP), ammonium (NH_4_^+^), nitrates (NO_3_^–^), phycocyanin-containing blue-green algae (BGA-PC), Chlorophyll-*a*, and Secchi depth.

Water samples for sequencing were taken from Lake Cajititlán using a Van Dorn type bottle and placed in plastic containers of 1 L capacity that were previously disinfected and washed. Two replicates of each sample were obtained, resulting in 2 L per sample and a total of 60 water samples. These samples were taken at the same sampling points, depth, and study period, as the measurements of physicochemical parameters. All of the samples were transported at 4°C to the Laboratory of Biotechnological Bioprocesses of Tecnológico de Monterrey at the Guadalajara campus for subsequent analyses.

### DNA Extraction, PCR Amplification, and Sequencing

To retain different microbial fractions, both water sample replicates were filtered independently using two different pore sized cellulose nitrate membranes (Whatman^TM^) connected to a vacuum pump. First, each replicate was filtered using a membrane with a pore size of 20–25 μm. Afterward, the obtained filtrate was passed through a second membrane with a pore size of 0.45 μm. Therefore, in total, two membranes of different pore sizes were obtained per replicate. Each of these two filters was then separately cut into pieces using sterile scissors and 100 mg of each were weighed and added to a lysing matrix to perform a DNA extraction and purification of the samples using the FastDNA Spin Kit for Soil (MP Biomedicals, OH, United States), according to the manufacturer’s instructions. The concentration of purified DNA was measured using a NanoDrop ND-1000 UV–Vis spectrophotometer (NanoDrop Technologies, Wilmington, DE).

To understand the abundance/composition of the cyanobacteria and microalgae communities present in Lake Cajititlán, PCR amplification was carried out separately for prokaryote vs. eukaryote identification. For prokaryotes, a *ca.* 460 bp fragment covering the V3–V4 hypervariable regions of the 16S rRNA gene was PCR amplified following the Illumina protocol for 16S Metagenomic Sequencing Library Preparation (Amplicon et al., 2013). For eukaryotes, a *ca.* 470 bp fragment of the V4 region of the 18S rRNA gene was amplified with primers previously shown to preferentially amplify microalgae (forward 5’-CCAGCASCYGCGGTAATTCC-3’ and reverse 5’-ACTTTCGTTCTTGATYRATGA-3’; Tragin et al., 2017). PCR products were run on a 1% agarose gel in a TAE buffer and visualized by GelRed staining (Biotium, United States) under UV light. A nested PCR was then performed to attach the dual indices and Illumina sequencing adapters using the Nextera XT Index kit (Illumina^®^), and electrophoresis was performed with the PCR products (1% agarose gel) to confirm that indexes and adapters were successfully attached to the libraries. A clean-up of the sequencing libraries was carried out with magnetic beads from the AMPure XP kit (Beckman Coulter) to later quantify using a Qubit 2.0 fluorometer (Life Technologies, Invitrogen^®^).

To achieve maximum operational efficiency in the Illumina sequencing platform, a single sequencing run was performed for both prokaryotic and eukaryotic 96-sample libraries combined in a single prep-plate and uniquely indexed (Amplicon et al., 2013). This was carried out by combining the prokaryotic and eukaryotic amplified products per sample, using a ratio of 70:30 prokaryotic to eukaryotic PCR product concentration, respectively. For high-throughput sequencing (2×300 bp, paired-end), the 96 samples were pooled at a concentration of 8 pM and loaded together with 30% Phix control into an Illumina^®^ MiSeq sequencer using the MiSeq Reagent Kit v3 (Illumina, San Diego, CA, United States) in the sequencing facilities of Tecnologico de Monterrey, Campus Monterrey. The sequencing run has been uploaded to the NCBI Sequence Read Archive with accession numbers PRJNA626359 (16S rRNA gene sequences) and PRJNA626364 (18S rRNA gene sequences).

### Bioinformatic Analyses

For sequencing data analyses, 16S rRNA and 18S rRNA gene sequences were split using the primer sequences as a criterion for division on the Galaxy open-source platform (Afgan et al., 2018). Once prokaryotic and eukaryotic sequences were separated, these were analyzed in the software QIIME 2.0 (Quantitative Insights into Microbial Ecology; Bolyen et al., 2019) following a standard bioinformatics pipeline. First, raw reads were demultiplexed and denoised into amplicon sequence variants (ASVs) using DADA2 (p-trim-left 0, p-trunc-len 440 nts). Afterward, two characteristics tables [FeatureData(Sequence) and FeatureData(Taxonomy)] were constructed using 99% similarity, with the SILVA version 132 and RDP version 11 databases used for 16S rRNA and the PR^2^ version 4.12.0 database used for 18S rRNA (Cole et al., 2013; Guillou et al., 2013; Quast et al., 2013; Yilmaz et al., 2013; del Campo et al., 2018). Then, the classifier was trained using the primers and the length of the samples through the Naives Bayes classifier method. Finally, taxonomic classification was performed with classify-sklearn and the file of the denoised sequences together with the trained classifier (Bolyen et al., 2019). Taxa bar plots were generated to assign the corresponding taxonomy to the ASV table, which were downloaded in CVS format from view.qiime2.org to continue further analysis.

### Statistical Analyses

To understand the effects of climatic conditions during the sampling period (2018) as well as over a period of 21 years (1998–2019), CONAGUA datasets were used to construct box plots comparing rainfall, evaporation rates, and maximum and minimum temperatures (CONAGUA Gobierno de México, 2020). A box plot of the TN:TP relationship was also constructed to better understand the limiting nutrient in Lake Cajititlán throughout the sampling year (2018) and during a 10 year history (2009–2019). In the case of tropical lakes/reservoirs, a ratio higher than 9 indicates a phosphorus-limited body of water, while a ratio lower than 9 represents nitrogen limitation (Salas and Martino, 2001). Likewise, a boxplot was constructed to depict the yearly behavior of the ecosystem-specific water quality index calculated through an algorithm from a previous report (Gradilla-Hernández et al., 2020b). Additionally, physicochemical parameters were analyzed spatially and temporally, and box plots were created.

Sequencing depth of the 16S and 18S rRNA genes was represented by a rarefaction curve performed in R by the rarefy function based on Hurlbert’s (1971) formulation, and the standard errors were based on Heck et al. (1975). For the following analyzes, only the taxonomic information of the cyanobacterial and microalgae communities was used. Read numbers were normalized using the package DESeq2 (Anders and Huber, 2010). To visualize, analyze and compare the information, bar plots of relative read abundance were performed using the Scale package. Taxa with proportions <0.01% were grouped as “others” and unclassified genera (cyanobacteria) or families (microalgae) were denoted by “Un” and the previous taxonomic level identified. Alpha diversity indices of Shannon (diversity), Simpson (proportional abundance), and Chao1 (microbial richness) were calculated using the diversity (Shannon and Simpson) and estimateR (Chao1) functions (Fisher et al., 1943; Cori et al., 2013), which were presented in a boxplot to observe the spatial and temporal variation within these diversity indices (Miller, 1981; Yandell, 1997).

Furthermore, changes in microbial community structure were analyzed by principal coordinate analysis (PCoA) using the cmdscale function in the vegan package, based on Bray-Curtis distances (Oksanen et al., 2016). In addition, using the Bray and Curtis dissimilarity index, permutational multivariate analysis of variance (perMANOVA) (*P* < 0.05) and analysis of similarity (ANOSIM) were used to test statistically significant differences in phytoplankton community composition at the spatial scale of Lake Cajititlán and temporal form (Clarke, 1993; Anderson, 2001; Anderson et al., 2006; Oksanen et al., 2016).

Unless stated otherwise, all statistical analyses were conducted with R version 3.5.3 (R Core Team, 2019) using the vegan package (Oksanen et al., 2016). Box plots and line diagrams were built using the ggplot2 package (Wickham, 2009). All boxplots were prepared to include the results of one-way analyses of variance (ANOVA) (α = 0.05) and Tukey’s HSD tests to determine significant differences.

### Detection and Quantification of Total Microcystin Content

For microcystin analysis, additional water samples were obtained from the same sampling sites and depths on July 15, 2019. This is historically the month that many fish die in Lake Cajititlán (Alatorre, 2015). This month has also been reported to present the lowest values of the water quality index specific for Lake Cajititlán, as reported by Gradilla-Hernández et al. (2020b), for a period of 9 years (Figure 2F).

Duplicate water samples were collected using a horizontal “Grab” or Van Dorn type bottle and placed in 500 mL high-density polyethylene wide-mouth bottles. All samples were transported at 4°C to the molecular microbiology laboratory of CIATEJ (Centro de Investigación y Asistencia en Tecnología y Diseño del Estado de Jalisco, A.C.) and were processed within 24 h.

Quantitative measurement of total microcystin content was carried out in duplicate by an enzyme-linked immunosorbent assay (ELISA) with microcystin specificity, using a commercial kit and following the manufacturer’s protocol (Prod. No. ALX-850-319, Enzo Life Science Inc. Farmingdale, United States). In accordance with the instructions and recommendations described by the manufacturer (Fischer et al., 2001), the cell lysis procedure of the samples was performed by freezing, thawing and sonication methods. Optical density values were measured at 450 nm using a Cytation^TM^ 3 (BioTek^TM^) microplate spectrophotometer, with a microcystin detection limit of 0.1 μg/L^–1^. Total microcystin concentrations of the samples were determined by interpolating a standard curve constructed with each run.

## Results

### Climatological Characterization and Water Quality Characteristics of the Lake Cajititlán

According to the historical behavior of climatological data, the climatological parameter that displayed the greatest variations during the rainy season was precipitation (Figure 2A), with July being the month that historically receives the highest rainfall, and consequently, an intensive runoff of pollutants to the lake. Tropical and subtropical lakes are more susceptible to excessive pollutant runoff, since the rainy season is very intense and changes in water quality parameters are reflected even faster in shallow and small lakes (Nobre et al., 2020). These trends are strongly associated with the lowest ES-WQI values observed during the month of July in Lake Cajititlán (Figure 2F), as well as with the greatest variations in the TN:TP ratio observed during the rainy season (Figure 2E).

The annual behavior of the TN:TP ratio uncovered temporal shifts triggered by the onset of the wet season (Figure 2E). The wet season (June–September) showed the highest values in June (>9) and then values close to 9 in July. These results suggest that Lake Cajititlán shifts from being phosphorus-limited at the beginning of the rainy season (June–July) and at the end of the rainy season (September), to being limited by nitrogen (<9) in August (Salas and Martino, 2001). These alternations are associated with the intensive runoff of fertilizers at the onset of the rainy season, increasing the TN:TP ratio during June and July, and then decreasing the ratio in August as a result of the consumption of nitrogenous compounds by the increasing phytoplankton communities (Hem, 1985; Raven et al., 1992; Cabello et al., 2009; Prangnell et al., 2019). Alternation between phosphorus and nitrogen limitation has been previously documented for tropical or subtropical lakes (Morris and Lewis, 1988). Figure 2E shows the mean annual behavior of the TN:TP ratio in Lake Cajititlán for the period (2009–2018).

**FIGURE 3:**
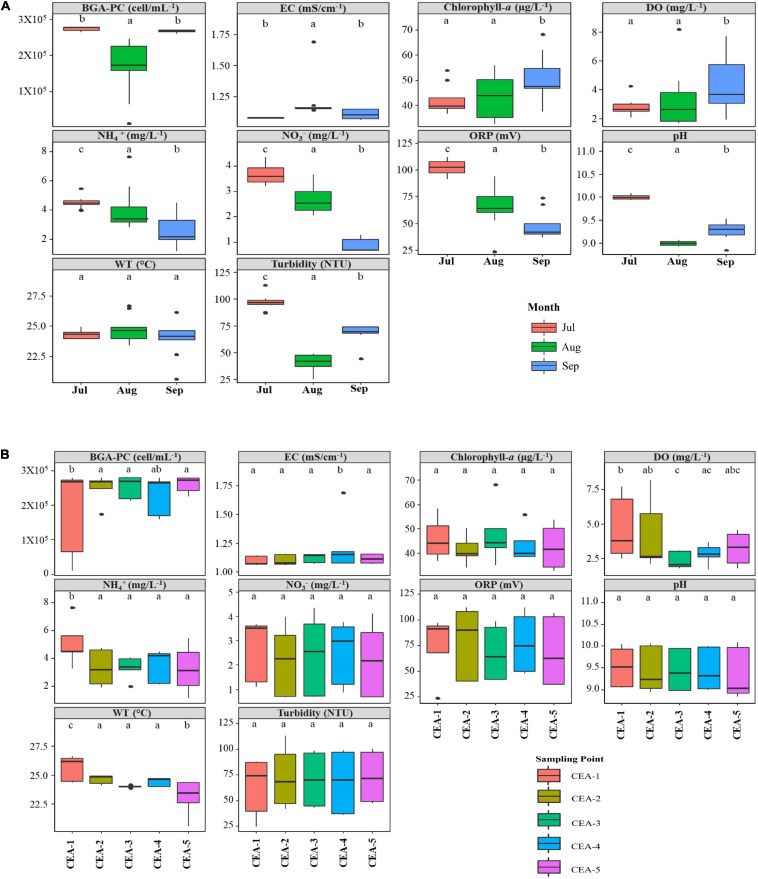
Physicochemical and environmental parameters by month and sampling site. **(A)** Box plots of physicochemical parameters by sampling month. **(B)** Box plots of physicochemical parameters by sampling point.

Similar to the changes historically observed for the behavior of the TN:TP ratio, the results of our monitoring program throughout the rainy season (July–September) indicated that most of the physicochemical parameters monitored showed significant temporal variations when comparing the values reported for the three different months (Figure 3A). NH_4_^+^, NO_3_^–^ and ORP were found to decrease over time (from July to September), whereas dissolved oxygen (DO) and Chlorophyll-*a* increased, indicating an increase in the photosynthetic activity in phytoplankton communities (Steel, 1980). Additionally, in small and shallow tropical lakes, such as Lake Cajititlán, very rapid and significant changes occur in the concentration of water quality parameters due to the effects of the rainy season (Nobre et al., 2020). In the case of Lake Cajititlán, the concentration of nutrients (NH_4_^+^, NO_3_^–^) were highest in July as a result of a concentration process due to evaporation during the hot-dry season (February–May) (Figure 2B) and due to the runoff of pollutants after the first rains. During the rainy season (August–September), the concentrations of nutrients were found to decrease as a result of a dilution process caused by high precipitation rates (Figure 2A). pH, turbidity, and BGA-PC displayed similar temporal patterns during the study period. Spatial analysis indicated significant variations in fewer parameters: WT, BGA-PC, EC, NH_4_^+^, and DO. DO displayed the most spatial variation, presenting hypoxic average concentrations (<2.0 mg L^–1^; Wu, 2009) at the two deepest analyzed sample sites (CEA-3 and CEA-5) (Figure 3B). Such DO levels (<5.0 mg L^–1^) are considered unsuitable for most aquatic organisms (Wu, 2009).

During the sampling months of this study, the water transparency was evaluated using a Secchi disc. Values ranged between 8.20 and 21.80 cm (Table 1). In an attempt to evaluate eutrophication in tropical areas, several indices (Lamparelli’s index or the Carlson’s Trophic State Index) that consider the particular characteristics of tropical environments have been developed. Some of these indices, however, do not consider water transparency, because this parameter is directly affected by the naturally high turbidity of tropical waters during most of the year, but especially during the rainy season (Carlson, 1977; Toledo et al., 1983; Salas and Martino, 1991; Lamparelli, 2004). However, if the results obtained from Secchi transparency and Chlorophyll-*a* of this study and the TP history database are compared with the Lamparelli’s index or the Carlson’s Trophic State Index, Lake Cajititlán is classified as hypereutrophic. This condition was previously reported by de Anda et al. (2019a) for Lake Cajititlán.

**TABLE 1:** Secchi depth by month and sampling site.

**Sampling point**	**Secchi depth (cm)**		
	**July**	**August**	**September**
CEA-1	9.5	36	8
CEA-2	7	15	8
CEA-3	10	17	9
CEA-4	8.5	23	8
CEA-5	10	18	8
Mean ± *SD*	9.00 ± 1.14	21.80 ± 7.57	8.20 ± 0.44

### Bioinformatic Analysis

Table 2 shows the number of reads obtained from 96 water samples collected from Lake Cajititlán. A total of 6,075,574 raw reads were sequenced for the hypervariable region V3–V4 of the 16S rRNA gene. From these, 42.71% were classified as bacteria using both the SILVA reference database and RDP, where cyanobacteria represented 0.45 and 0.53%, respectively, for each database. Unclassified reads represented 57.29% for SILVA and 57.24% for RDP. A total of 3,189,413 raw reads were sequenced for the V4 region of 18S rRNA gene and classified using the PR^2^ reference database. From these, 67% were classified as eukaryotic, and microalgae represented 61.74%. Unclassified reads represented 32.95% of total eukaryotic sequences.

**TABLE 2:** Number of reads obtained from sequencing data analysis.

**Gene region**	**Raw reads**	**Classified reads**		**Unclassified reads**
		**SILVA**	**RDP**	
V3–V4 16S rRNA	6,075,574	Bacteria: 2,594,766 Cyanobacteria: 11,732 (0.45%)	Bacteria: 2,597,984 Cyanobacteria: 13,741 (0.53%)	Silva: 3,480,808 RDP: 3,477,590

		**PR^2^**		

V4 18S rRNA	3,189,413	Eukaryotes: 2,138,457 Microalgae: 1,320,388 (61.74%)		1,050,956

**FIGURE 4:**
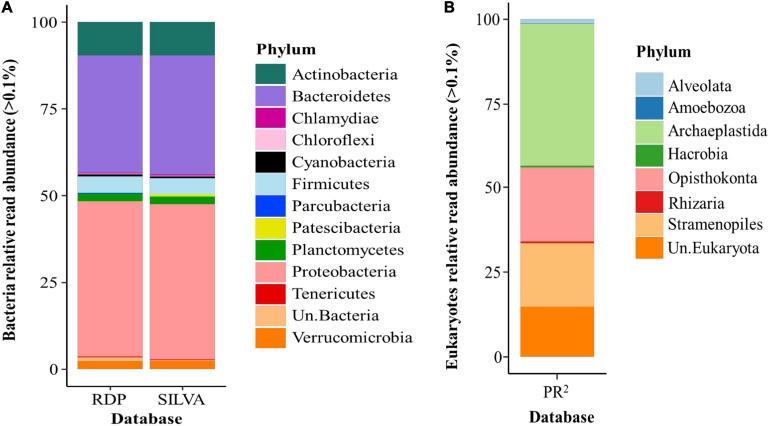
Bacteria and eukaryotic phyla relative read abundance from different databases. **(A)** Relative read abundance of bacteria phyla from different databases (RDP and SILVA). **(B)** Relative read abundance of eukaryotes from PR2 database.

Figure 4A shows the general results of the bacteria taxonomic annotation pipeline, in which very similar taxonomic classification results (bacterial phyla) were obtained using SILVA vs. RPD (Figure 4A and Supplementary Table S1). The three most abundant phyla were Proteobacteria (SILVA: 44.79%, RDP: 44.89%), Bacteroidetes (SILVA: 34.42%, RDP: 33.92%) and Actinobacteria (SILVA: 9.54%, RDP: 9.62%). Although the abundance and composition of bacteria were very similar in both databases, some differences can be observed for Parcubacteria (0.18%, found only through RDP) and Patescibacteria (0.38%, found only in SILVA). This proportion of heterotrophic bacteria is only presented as a comparison to the proportion of cyanobacteria present in Lake Cajititlán in the different databases. The highest proportion of reads classified as cyanobacteria were found in the RDP database (0.52%); therefore, for subsequent bioinformatic analysis, only these cyanobacteria data were used.

The analysis of relative read abundance of eukaryotes shows that Archaeplastida (42.20%), Opisthokonta (22.08%), and Stramenopiles (19.17%) phyla were the most abundant in Lake Cajititlán during this study, which contain some taxonomic groups of microalgae (Supplementary Table S2). Some microalgae can also be grouped in the less abundant eukaryotic phyla, such as Aleovata (1.29%) and Hacrobia (0.28%) (Figure 4B and Supplementary Table S2). The rarefaction curve shows that samples reached an asymptote (Supplementary Figure S1).

### Spatial and Temporal Variations of the Diversity and Abundance of Phytoplankton Communities

**FIGURE 5:**
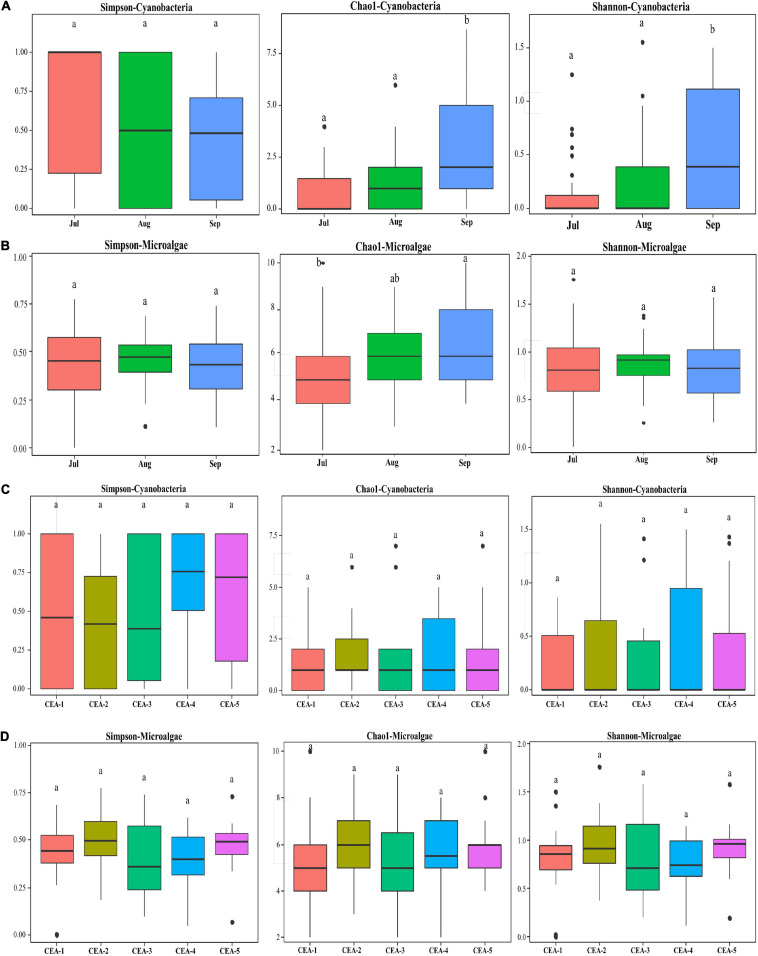
Box plots for diversity and richness indices of the cyanobacterial and microalgae communities in different months and sampling sites. **(A)** Cyanobacterial communities by sampling month. **(B)** Microalgae communities by sampling month. **(C)** Cyanobacterial communities by sampling site. **(D)** Microalgae communities by sampling site.

Richness and diversity of cyanobacterial and microalgal communities at different sites and sampling months were assessed using the Chao1, Simpson and Shannon-Weaver indices (Carey et al., 2013; Wu et al., 2019; Figure 5). The richness estimated by Chao1 and the diversity indicated by the Shannon index showed significant increases in the cyanobacterial communities during the study period (from July to September), whereas no differences were shown by the Simpson index (Figure 5A and Supplementary Table S3). Microalgae richness also significantly increased from July to September; however, diversity remained unchanged (Figure 5B and Supplementary Table S5). Changes in precipitation and increased nutrient runoff were observed, as reported for other tropical subtropical lakes (Moss et al., 2011; Havens and Jeppesen, 2018). There were no significant spatial variations for cyanobacteria or microalgae communities (Figures 5C,D and Supplementary Tables S4, S6). Shallow lakes regularly display a polymictic character with complete mixing events during summer, mainly due to precipitation and wind, which results in destratification and complete mixing of the water column (do Nascimento-Moura et al., 2012; Kerimoglu and Rinke, 2013; Cavicchioli et al., 2019; Gradilla-Hernández et al., 2020a).

**FIGURE 6:**
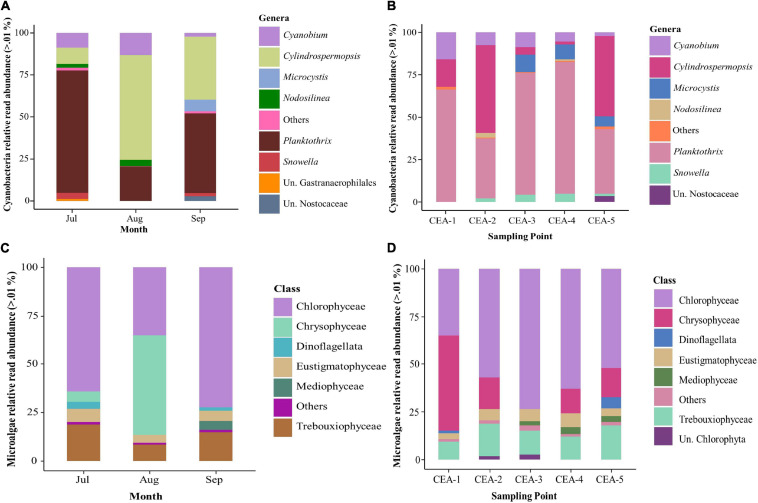
Relative read abundance of cyanobacterial and microalgae communities by month and sampling point. **(A)** Cyanobacteria genera relative read abundance by sampling month. **(B)** Cyanobacteria genera relative read abundance by sampling point. **(C)** Microalgae classes relative read abundance by sampling month. **(D)** Microalgae classes relative read abundance by sampling point.

According to relative read abundance, *Planktothrix* and *Cylindropermopsis* were consistently the most abundant cyanobacterial genera across all sampling sites (58.01% and 24.43, respectively), and months (47.37 and 37.80%, respectively), of this study, which have been dominant in other tropical or subtropical lakes studies (Figures 6A,B and Supplementary Tables S7, S8; Jöhnk et al., 2008; Gallina et al., 2011; Michalak, 2016; Barros et al., 2017; Guellati et al., 2017). Other groups that were not able to be classified at the genus level were Un. Gastranaerophilales and Un. Nostocaceae. Any groups of cyanobacteria with less than 0.01% relative read abundance were classified as “others.”

For microalgae communities, it was not possible to classify the reads at the genus level, and therefore, they were analyzed at the class level. The top two most abundant microalgae classes were Chlorophyceae and Chrysophyceae. Chlorophyceae was the most abundant at all sampling sites and months (57 and 57.12%, respectively), except for the month of August, when Chrysophyceae was the dominant class (51.55%) (Figures 6C,D and Supplementary Tables S9, S10). Chlorophyceae is a dominant class in tropical eutrophic shallow lakes, whereas Chrysophyceae is considered characteristic of oligotrophic and mesotrophic waters (Kristiansen, 1985; Chellappa et al., 2008; Silva et al., 2014). However, its dominance in subtropical and eutrophic water bodies has been detected during the summer season, with its presence being associated with the addition of allochthonous nutrients (Munch, 1980; Shi et al., 2019).

**FIGURE 7:**
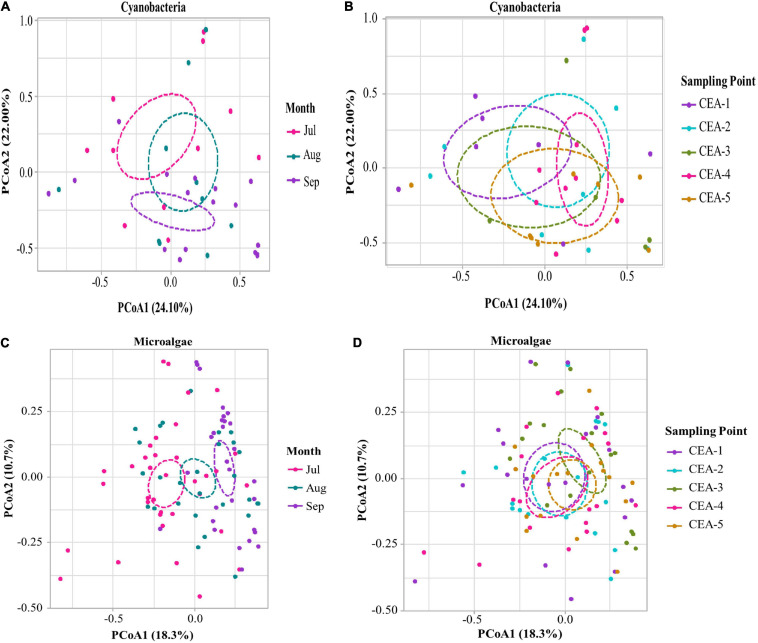
Principal coordinates analysis (PCoA) of the dissimilarities among microbial communities by month and sampling point using the Bray-Curtis distances. **(A)** PCoA of cyanobacterial communities by sampling month. **(B)** PCoA of cyanobacterial communities by sampling point. **(C)** PCoA of microalgae communities by sampling month. **(D)** PCoA of microalgae communities by sampling point.

To analyze spatial and temporal changes in the composition of cyanobacteria and microalgae communities, a principal coordinate analysis (PCoA) was performed based on Bray-Curtis distances (Figure 7). The temporal analysis of cyanobacteria (ANOSIM *R* = 0.0931, *P* = 0.005) and microalgae (ANOSIM *R* = 0.1209, *P* = 0.001) reflected a temporary transition between the months of July and September (Figures 7A,C and Supplementary Table S1). In tropical regions, this behavior reflects the seasonal climatic changes, which alters rainfall and the biogeochemical processes that makes nutrients available to a greater extent for these communities (Sarmento et al., 2013; Steffen et al., 2015; Greaver et al., 2016). Similar community compositions of cyanobacteria (ANOSIM *R* = −0.02209, *P* = 0.249) and microalgae (ANOSIM *R* = −0.01543, *P* = 0.280) were observed at all sampling points (Figures 7B,D and Supplementary Table S1).

To analyze the spatial and temporal changes in the composition of the cyanobacterial and microalgal communities, a principal coordinate analysis (PCoA) based on the Bray-Curtis distances was performed (Figure 7). The temporal analysis of cyanobacteria (ANOSIM *R* = 0.0931, *P* = 0.005) and microalgae (ANOSIM *R* = 0.1209, *P* = 0.001) revealed a temporal transition between the months of July and September with 55.67% of the variance total explained by the three main eigenvalues for cyanobacteria and 35.1% for microalgae (Supplementary Tables S11–S13). In cyanobacteria, the first two eigenvalues explained 24.10 and 22% of the total variation in the data during the sampling months, while in microalgae, the first two eigenvalues explained 18.36 and 10.79% of the total variation (Figures 7A,C). In tropical regions, this behavior reflects seasonal climate changes, which alter rainfall and biogeochemical processes that make nutrients more available to these communities (Sarmento et al., 2013; Steffen et al., 2015; Greaver et al., 2016). Similar community compositions of cyanobacteria (ANOSIM *R* = −0.02209, *P* = 0.249) and microalgae (ANOSIM *R* = −0.01543, *P* = 0.280) were observed at all sampling points (Figures 7B,D and Supplementary Table S11).

### Quantification of Total Microcystin Concentration

The total microcystin content in the lake water samples is shown in Table 3. The lowest concentrations of this toxin (<0.15 μg/L) corresponded to the following sampling sites and depths: CEA-2 (1.9 m) and CEA-4 (2.9 m). Conversely, the highest concentrations of this toxin 0.880 and 0.750 μg/L corresponded to sampling sites CEA-1 (30 cm) and CEA-5 (30 cm), respectively. Moreover, the highest concentrations were detected on the surface of the lake, where one would expect to find the highest concentration of cyanobacteria, forming part of the algal blooms in Lake Cajititlán. One-way ANOVAs (α = 0.05) were performed to observe the difference in total microcystin concentration by site and sampling depth, both of which showed no significant differences (*P* < 0.05).

**TABLE 3:** Total microcystin concentration in water samples from Lake Cajititlán.

**Sampling site**	**Mean concentration of total microcystin at 30 cm depth (μg/L)**	**Mean concentration of total microcystin at maximum depth (μg/L)**
CEA-1	0.880	0.459 (1.4 m)
CEA-2	0.683	<0.15 (1.9 m)
CEA-3	0.210	<0.15 (3 m)
CEA-4	0.315	<0.15 (2.9 m)
CEA-5	0.750	0.330 (2.7 m)

**P-value by sampling depth was 0.05, P-value by sampling site was 0.39.**

## Discussion

### Climatological and Water Quality Characteristics of Lake Cajititlán

The present study revealed that the composition of the phytoplankton community in Lake Cajititlán displayed significant temporal changes during the study period caused by the rainy season (Figures 5, 7). Additionally, the findings of this study suggest that significant rainfall variations cause extreme seasonal surface runoff and rapid changes in the water quality (Chlorophyll-*a*, DO, NH_4_^+^, and NO_3_^–^) of this subtropical lake, as well as rapid variations in the phytoplankton community (Figures 3, 2, 5, 7). Studies on the temporal variations in the phytoplankton community have been carried out in temperate regions, but only a few have been reported in tropical or subtropical regions, such as Lake Cajititlán (Limón-Macias et al., 1983; Umaña-Villalobos, 2010; Riediger et al., 2015; Li et al., 2018; Ma et al., 2019; Quevedo-Castro et al., 2019). The wet season in tropical and subtropical areas exacerbates the cultural eutrophication of surface water bodies when there is intensive agricultural activity in their basin (Barbosa, 2009; Cunha et al., 2013). Tropical and subtropical water bodies are also susceptible to other anthropogenic sources of pollutants from urban areas (e.g., wastewater effluents) due to less efficient wastewater treatment facilities. Some causes of inadequate wastewater treatment in developing countries in tropical or subtropical regions are the lack of funds, restricted local budgets, and the lack of local expertise, leading to a deficit in the construction and satisfactory operation of treatment facilities (Paraskevas et al., 2002). These results coincide with the behavior of the TN:TP ratio and the water quality parameters (Chlorophyll-*a*, DO, NH_4_^+^, and NO_3_^–^), which presented greater variation during the rainy season and which directly affect the water quality index of the Cajititlán Lake.

Tropical and subtropical bodies of water show major changes in their water quality and biotic communities in response to eutrophication (Gillett et al., 2016). The pronounced wet season in these regions causes modifications in the physical and chemical characteristics of the water and highly influences phytoplankton dynamics (Figueredo and Giani, 2009). Furthermore, as a result of tropical and subtropical climatic conditions, the biomass production potential of phytoplankton, on a given nutrient basis, can be expected to be higher in tropical lakes than in temperate lakes (Lewis, 1974). Specifically, in lake Cajititlán, rainfall has been reported to cause significant changes in the concentrations of the main forms of dissolved inorganic nitrogen, such as NH_4_^+^ and NO_3_^–^. These nitrogenous compounds increase at the beginning of the wet season due to the surficial runoff containing high loads of nutrients, later to generate a dilution effect as the water level increases throughout the wet season (Gradilla-Hernández et al., 2020b). This is consistent with the results of the current study, as the concentrations of NH_4_^+^ and NO_3_^–^ were higher in the first sampling (July) (Figure 3A) and decreased through the rainy season (Gradilla-Hernández et al., 2020b). In this study and as reported by de Anda et al. (2019a), a high content of BGA-PC and Chlorophyll-*a* was detected (Figure 3).

Both phosphorus and nitrogen are essential elements for the growth of phytoplankton and for primary production (Carpenter et al., 1998). Phosphorus has been considered the most important nutrient in the control of phytoplankton in lakes at high latitudes, but in the case of tropical and subtropical regions, it has been suggested that nitrogen is the limiting nutrient in some cases (Vincent et al., 1984; Ramos-Higuera et al., 2008). This is expected as natural sources of phosphorus can be traced to the chemical weathering of rock, which is a thermally sensitive process that occurs at considerably higher rates where the temperature is higher (Meybeck, 1979). However, historically, Lake Cajititlán was more phosphorus-limited at the beginning (June–July) and at the end of the rainy season (September) and in the intermediate time (August), it appeared to be more nitrogen-limited (Figure 2F). In July, after the onset of the rainy season, there are many nitrogen sources of pollution that are carried to the lake by runoff. Rain episodes can be very intense in tropical or subtropical regions and result in heavy runoff of nitrogenous compounds into water bodies (Cunha et al., 2013). However, in August these forms of nitrogen decrease rapidly as there is an increase in the community of phytoplankton that consumes these compounds (Figure 3A). This indicates the intensification of the nitrification and denitrification processes. After cyanobacteria fix molecular nitrogen (N_2_), NH_4_^+^/NH_3_ are converted into nitrites (NO_2_) by a group of bacteria of the *Nitrosomes* genus, to be later converted to NO_3_^–^ by bacteria of the genus *Nitrobacter*, which are further metabolized by aquatic plants and algae (Hem, 1985; Raven et al., 1992; Cabello et al., 2009; Prangnell et al., 2019).

Cyanobacteria not only have the ability to fix N_2_ but also have the ability to assimilate nitrogen from a number of N-containing compounds, such as NH_4_^+^, NO_3_^–^, NO_2_, and urea. In fact, several experimental and *in situ* studies have shown that cyanobacteria appear to outcompete other phytoplankton species for reduced forms of N (Blomqvist et al., 1994; Ferber et al., 2004; Flores and Herrero, 2005; Cronberg and Annadotter, 2006; McCarthy et al., 2009). This information is consistent with the large increase of cyanobacteria compared to microalgae observed in this study (Figures 5A,B). One of the symptoms of degraded water quality is the increase of phytoplankton biomass as measured by the concentration of Chlorophyll-*a*. Chlorophyll-*a* concentrations are often higher after rainfall, particularly if the rain has flushed nutrients into the water. Receiving waters with high levels of nutrients from fertilizers, septic systems, sewage treatment plants, and urban runoff may have high concentrations of Chlorophyll-*a* and high amounts of phytoplankton (Monbet, 1992; Hinga et al., 1995; Ward et al., 1998; Wellman et al., 2002; Brando et al., 2006; Scanes et al., 2007). In this study, an increase of Chlorophyll-*a* was observed (July to September), indicative of nutrients being flushed into the lake during the rainy season (Figure 3A).

In addition to being aesthetically unpleasant, cyanobacterial blooms manifest as a reduction in water transparency that can inhibit the growth of aquatic macrophytes due to limited light penetration; this subsequently disrupts invertebrate and fish habitats (Scheffer et al., 1993; Li, 1998; Paerl et al., 2001; Pick, 2016). Furthermore, combined wastewater/rainfall enters Lake Cajititlán without any treatment, because WWTPs do not have separate pipes for wastewater vs. rainfall water (de Anda et al., 2019a; Gradilla-Hernández et al., 2020a). This is reflected in the results of the Secchi depth measurements and in the higher values of NH_4_^+^ and NO_3_^–^ in July (Table 1 and Figure 3A). In August, the water transparency of Lake Cajititlán improved, probably due to the dilution effect generated by the rains (Martinez-Urtaza et al., 2004). However, the Secchi depth decreased again in September, which could indicate that due to the high availability of nutrients in the lake, growth of the phytoplankton community may be triggered, as observed in the increase in Chlorophyll-*a* throughout the study (Figure 3).

### Spatial Stability of the Phytoplankton Community

A previous study on Lake Cajititlán reported spatial variations for these physicochemical parameters—pH, NO_3_^–^ and NO_2_^–^ (Gradilla-Hernández et al., 2020a). The authors correlated these variations with the configuration of the lake, since CEA-2 to CEA-4 are at the center of the lake, while CEA-1 and CEA-5 are at the west and east sides, respectively. This current study is consistent with the results of that previous study, as only a few parameters, BGA-PC, NH_4_^+^, and WT, gave the most significant variations, which were mainly found at the CEA-1 sampling site (Figure 3B). This sampling site is the closest to the San Miguel Cuyutlán WWTP (Figure 1), which is the plant with the highest capacity (60 L/s) and processes the largest volume of municipal wastewater, which it discharges directly into the lake without tertiary treatment (Gobierno del Estado de Jalisco, 2014; de Anda et al., 2019a). Likewise, in the CEA-1 sampling site, a higher dilution range of BGA-PC was found, which suggests that the WWTP point-source pollution favored the development of this population. Municipal wastewater can contain nitrogen and phosphorus compounds from human waste as well as from soaps and detergents. This condition can facilitate the growth of cyanobacteria in water bodies if these nutrients are not properly eliminated (Rapala and Sivonen, 1998; Gomes de Quevedo and Da Silva, 2016).

### Dynamics and Abundance of the Dominant Taxa of Lake Cajititlán

The genera *Planktothrix* and *Cylindrospermopsis* were the dominant groups of cyanobacteria (Figure 6). This finding is similar to the results found in other freshwater studies, which report the dominance of any of these two genera (Lira et al., 2011; Bonilla et al., 2012; Barros et al., 2017; Guellati et al., 2017). *Pl*a*nktothrix* is one of the most important microcystin-producing genera in temperate lakes (Fastner et al., 1999; Vasas et al., 2013). However, very few articles have reported *Planktothrix* as a predominant genus for tropical and subtropical areas (Reynolds et al., 2002; Jöhnk et al., 2008; Gallina et al., 2011; Michalak, 2016; Barros et al., 2017; Guellati et al., 2017).

Within the *Planktothrix* genus, it is frequently the *P. agardhii* and *P. rubescens* species that dominate the phytoplankton community in the water column (Kurmayer et al., 2016). *P*. *agardhii* and *P*. *rubescens* are known to be the most efficient light harvesters within the phytoplankton community, which is, in part, due to their possession of specific accessory pigments, namely the phycobilins (phycoerythrin, phycocyanin (BGA-PC), and allophycocyanin) (Kurmayer et al., 2016). The red-pigmented phycoerythrin (PE)-rich genotypes are found in *P. rubescens*, while the green-pigmented phycocyanin (PC)-rich genotypes are frequently found in *P. agardhii* (Komárek and Komárková, 2004). In this study, only the BGA-PC phycobilin was analyzed (Figure 3A), and it was mainly found to be associated with the *P. agardhii* species, since *Planktothrix* was the most abundant genus. In addition, Lake Cajititlán is located in a subtropical region, which contains environmental conditions that are advantageous for *P. agardhii*, since it has been reported in shallow and subtropical lakes (Baker and Humpage, 1994; Kruk et al., 2002; Suda et al., 2002; Bouchamma et al., 2004).

The genus *Cylindrospermopsis* was the second most abundant in this study, and the most abundant during August (Figure 6A). This genus was originally found only in tropical and subtropical regions, but it has now expanded into temperate areas (Haande et al., 2008). In México there are few reports of this cyanobacteria. *C. raciborskii* is one species that has been identified (Komárek and Komárková-Legnerová, 2002; Valadez et al., 2005). Most of the studies on *C. raciborskii* have been carried out on reservoirs used for agriculture or recreation, and in some cases on drinking water sources (Lei et al., 2014; Menezes et al., 2020). Agriculture is one of the main activities in Lake Cajititlán, so it is possible that the abundance of this bacterium is associated with the increase in the concentration of nutrients from fertilizers, mainly during the rainy season, which enhances the runoff of nutrients to the lake. This effect has been observed in several subtropical studies (Munch, 1980; Li et al., 2010; Shi et al., 2019).

In this study, Chlorophyceae (54.02%), Chrysophyceae (23.42%), and Trebouxiophyceae (13.24%) were the most representative microalgal taxonomic classes, suggesting that these microalgae families could be participating in the algal blooms observed in Lake Cajititlán. Andrade and Giroldo (2014) reported similar results in a shallow subtropical lake, where they found Chlorophyceae (30.8%) and Bacillariophyceae (24.8%), followed by Cyanophyceae (13.6%), Chrysophyceae (9.3%), Euglenophyceae (7.0%), and Zygnematophyceae (6.5%) to be the most represented taxonomic classes in terms of richness. In some lakes of volcanic origin in Mexico, the presence of Trebouxiophyceae has been reported; these lakes are also endorheic, and they are located in regions where the rainy season runs from June to September (Instituto de Geografía UNAM, 1990; Godínez-Ortega et al., 2017).

Shallow lakes such as Lake Cajititlán, generally exhibit fluctuations between various phytoplankton communities (Nixdorf et al., 2003). A phytoplankton community is considered to be in steady state when (i): one, two or three species contribute more than 80% of the biomass; (ii) their existence or coexistence persists for more than 2 weeks; and (iii) the total biomass does not increase significantly during the period analyzed (Sommer et al., 1993). In this study, a clear steady state was observed in the cyanobacteria community. The most abundant genera found in this study, *Planktothrix and Cylindrospermopsis* (at 80.58%), persisted and coexisted throughout the study period (Figure 6A). Some studies have shown that an equilibrium condition with cyanobacterial dominance could be expected in some steady state environments. However, several factors can influence the prevalence of cyanobacteria in phytoplankton communities, such as the water temperature, the mixing of the water column, the rainfall, and the limiting nutrients, among others (Figueredo and Giani, 2009). Similarly, Nixdorf et al. (2003) reported a steady state condition dominated by *Planktothrix* in shallow eutrophic lakes. Regarding the microalgae classes, a steady state could not be established because these classes were not constant in abundance during the study (Figure 6C).

The TN:TP ratio can be used to understand the dynamics of N and P in the aquatic system, and it can likewise be employed as a diagnostic criterion to assess the type of phytoplankton found under different nutrient, namely nitrogen and phosphorus, concentrations (Smith, 1983; Gržetić and Camprag, 2010). The trend in the relative abundance of *Planktothix* and *Cylindrospermopsis* (Figure 6A) contrasted with the trend of the TN:TP ratio (Figure 2E), uncovering possible relationships between the TN:TP ratio and the relative abundances of specific cyanobacteria populations. When phosphorus was found to be the limiting nutrient (July and September), the abundance of the *Planktothix* genus was higher, and when the limiting nutrient was nitrogen, *Cylindrospermopsis* presented a higher abundance. Interestingly, *Cylindrospermopsis* is known to be found in higher abundances in tropical lakes, where nitrogen is commonly the limiting nutrient (Talling and Lemoalle, 1998; Haande et al., 2008) and *Planktothix* is frequently found in temperate and mesotrophic lakes where phosphorus limitation is common (Welch and Naczk, 1992; Orr and Jones, 1998; Fastner et al., 1999; Vasas et al., 2013).

### Fish Mortality in Lake Cajititlán

Based on Lake Cajititlán’s seasonal characterization and water quality characteristics presented herein and in previous reports (Gradilla-Hernández et al., 2018, 2020a,b; de Anda et al., 2019a,b), the problem of fish kills is attributable to the eutrophication problem of this reservoir. Eutrophication is enhanced during the rainy season, due to an increase in the concentration of nutrients from fertilizers, sewage treatment plants and urban runoff, triggering a surge in the growth of phytoplankton communities (Figures 3, 2, 5). In this study, the lowest DO concentrations were recorded during August when concentrations below 2 mg/L^–1^ were measured. These low DO concentrations may be related to wastewater discharge from the WWTPs or to the enrichment of nutrients of the lake through surficial runoff. This is consistent with the fact that July and August displayed the highest levels of nutrients (NH_4_^+^and NO_3_^–^), which could favor the growth of phytoplankton populations (Figure 3A). DO concentrations of 5 mg/L or more are suitable for most aquatic organisms, and concentrations below 2 mg/L are considered hypoxic (Wu, 2009). Possible signs of a fish kill due to oxygen depletion are sluggish fish movements, fish gasping at the surface, larger fish dying earlier than smaller fish of the same species, and episodes occurring at night or in the early morning, as DO varies significantly between day and night in eutrophic water bodies (Helfrich and Smith, 2010; Nguyen et al., 2016). This diurnal phenomenon commonly occurs in eutrophic lakes, because, during the day, the intensity of radiation increases photosynthetic activity, while at night, DO is reduced significantly through respiration of the phytoplankton community, and release of carbon dioxide (CO_2_). When the CO_2_ levels in water become too high, fish have difficulty obtaining sufficient oxygen from the water, resulting in suffocation and death (Mallya and Thorarensen, 2007; Nguyen et al., 2016). This process was reported by Gradilla-Hernández et al. (2020a) for Lake Cajititlán, where significantly lower values of dissolved oxygen were found during nocturnal monitoring. During the sampling process of the present study, fish were observed gasping at the surface and dead fish were also seen floating (Supplementary Figure S2). These events were documented at 7 a.m. on July 15, 2019. Therefore, the results found in this study are consistent with what had been previously established: that fish kills in Lake Cajititlán during the rainy season could be related to a decrease in water quality, resulting in an increase in phytoplankton communities, leading to the depletion of DO in the water.

Although there is strong evidence to suggest that the fish kills in Lake Cajititlán have resulted mainly from anoxia, in this study, we investigated the microcystin concentration as another factor that could be associated with these events. Two of the main genera of microcystin-producing cyanobacteria (*Microcystis* spp., and *Planktothrix* spp.) were detected (Kaebernick and Neilan, 2001; Gupta et al., 2003). The highest concentration of microcystin detected in Lake Cajititlán was 0.880 μg/L. Concentrations <1 μg/L have may not stress fish but could influence their behavior and could impair development (Bury et al., 1996; Baganz et al., 1998; Wiegand et al., 1999). However, there are studies that report harmful effects (including mortalities) in fish at different life stages at microcystin concentrations ranging from 0.08 to 500 μg/L (Oberemm et al., 1997, 1999; Ernst et al., 2001; Liu et al., 2002). Additionally, several researchers have analyzed the content of microcystin in fish tissues and have reported that there is accumulation of microcystin in different tissues, which can cause reduction in the embryonic development of fish, as well as pathophysiological, histological, and ultrastructural damage (Bury et al., 1997; Jos et al., 2005; Prieto et al., 2006; Zhang et al., 2007; Atencio et al., 2008; Papadimitriou et al., 2012). Unfortunately, the content of this toxin in fish tissues was not analyzed, so it is necessary to carry out further studies to determine if there is bioaccumulation and possible toxicological effects.

Fish kills can severely reduce the productivity of recreational and commercial fisheries, and as a result, economic loss can be substantial. The eutrophication problem in Lake Cajititlán has affected fishing activity with the death of thousands of fish in recent years (Alatorre, 2015). The local fishermen have limited economic opportunities without this activity (Velázquez-López et al., 2012).

## Conclusion

This study revealed that the composition of the phytoplankton community in Lake Cajititlán changed rapidly and that the population increased throughout the study period. These trends were the result of the rainy season, which causes high seasonal surface runoff and therefore rapid changes in the water quality (Chlorophyll-*a*, DO, NH_4_^+^ and NO_3_^–^). Within the cyanobacterial community, it was found that *Planktothrix* and *Cylindrospermopsis* were the dominant genera of the cyanobacterial community, while the Chlorophyceae, Chrysophyceae, and Trebouxiophyceae classes where the most abundant within the microalgae community. Some species of the genus *Planktothrix* are considered potentially toxic because they have the capacity to produce microcystins, which were detected in this study between 0.210 and 0.880 μg/L^–1^. Finally, the TN:TP ratio presented an alternating trend indicating that Lake Cajititlán is limited by phosphorus at the onset and at the end (July and September, respectively), of the rainy season, and more nitrogen-limited during the intermediate month (August) of this season. The trend observed in the relative abundance of *Planktothix* and *Cylindrospermopsis* (Figure 6A) contrasted with the trend of TN:TP ratio (Figure 2E), uncovering possible relationships between the TN:TP ratio and the relative abundances of specific cyanobacteria populations.

The evidence presented in this study showed that the death of fish in Lake Cajititlán could be related mainly to anoxia, caused by the rapid changes in DO levels as a result of phytoplankton blooms. These blooms were the result of nutrients entering the lake through runoff during the rainy season. Special attention should be given to small and shallow endorheic lakes located in subtropical areas. In these systems, seasonal rainfall events result in large inputs of pollutants into freshwater systems, which in turn rapidly affect the phytoplankton community.

Although monthly sampling revealed relevant trends and patterns in the joint behavior of the TN:TP ratio, the climatic conditions, and the phytoplankton abundance data during the rainy season (especially when analyzing the relative abundance of *Planktothix* and *Cylindrospermopsis)*, future studies are warranted to confirm and deepen the understanding of the dynamics of these communities through weekly sampling. More frequent sampling of both abundance and of water quality, in addition to daily or weekly measurements of climatic variables would strengthen what is known regarding the changing behavior of subtropical water bodies during the rainy season, specifically those that are strongly affected by anthropogenic activity. To improve the characterization of microbial taxonomic level, future studies will be carried out by shotgun sequencing of selected samples. This will provide a more comprehensive understanding of the microbial communities in Lake Cajititlán, thus shedding light onto the metabolic changes that may be occurring, which allow these microorganisms to adapt to their environment.

## Data Availability Statement

The authors acknowledge that the data presented in this study must be deposited and made publicly available in an acceptable repository, prior to publication. Frontiers cannot accept a manuscript that does not adhere to our open data policies.

## Author Contributions

OD-T, MG-H, HS, and CS-G: conceptualization. OD-T and MG-H: data analysis. MG-H, CS-G, OL-M, DO-N, and AP: funding acquisition. OD-T, MG-H, JA, AP, and CS-G: methodology. MG-H and CS-G: project administration. MG-H, AP, OL-M, and CS-G: resources. All authors wrote and approved the manuscript.

## Conflict of Interest

The authors declare that the research was conducted in the absence of any commercial or financial relationships that could be construed as a potential conflict of interest.
